# Optimization and accuracy analysis of track straightness measurement based on total station free station method

**DOI:** 10.1038/s41598-026-37100-1

**Published:** 2026-01-22

**Authors:** Dingliang Yang, Jingui Zou

**Affiliations:** 1 School of Civil Engineering and Architecture, Suqian University, No.399 South Yellow River Road, Wuchang District, Suqian City, 223800 China; 2 School of Geodesy and Geomatics, Wuhan University, No.299 Bayi Road, Wuchang District, Wuhan City, 430070 China

**Keywords:** Free station method, Deviation measurement, Measurement optimization, Track straightness, Total station, Engineering, Physics

## Abstract

Aiming at the problems that the traditional method of total station in high-precision track straightness measurement is greatly restricted by the on-site environment, and the error control is difficult, this study proposes the free station method of the total station to establish a nonlinear measurement point deviation calculation model. Based on the Monte Carlo simulation, the influences of different angular measurement errors, distance measurement errors, and total station erection positions on the accuracy of the free station method are systematically analyzed. To ensure the accuracy of the deviation of measurement points, high-precision angular measurement is the key. Optimizing the total station erection position can eliminate distance measurement errors. The total station is set up at 50 m and 100 m along the 160-meter track, respectively, with a vertical distance of 4 m from the track. The mean square error of deviation of most measurement points along the entire line is successfully controlled within ± 0.3 mm. This method can be widely applied in engineering fields such as high-speed railways, precision guide rails, and large-scale structural installations, where sub-millimeter-level position tolerance detection is required in complex environments.

## Introduction

Straightness error is a critical indicator for evaluating the geometric accuracy of precision mechanical guideways, shafts, and large structural components. Its accurate measurement is essential for ensuring the performance of machine tools, instruments, and high-end equipment^1,2^. Conventional methods such as straightedge, level, and taut wire techniques^3^ remain common but often lack efficiency or precision. With advances in electro-optics, high-precision methods based on laser alignment^4^, laser interferometry^5^, and autocollimation^6^ have become mainstream. These techniques establish a stable optical reference and detect deviations directly using PSD or CCD sensors^7,8^, greatly enhancing measurement capabilities. Furthermore, dedicated sensing systems have been developed for specialized applications including deep-hole inspection^9^, seamless steel tube measurement^10^, and online monitoring^11^. However, their application to long-range (> 100 m) track measurement in complex, obstructed field environments remains challenging due to stringent requirements for stable reference lines and clear line-of-sight. Conversely, traditional total station methods offer flexibility but lack a systematic framework to achieve and guarantee sub-millimeter accuracy in such scenarios, as their accuracy is highly sensitive to station placement, which is often chosen empirically.

Measurement accuracy is affected by various error sources, making error analysis and compensation a research priority. Instrument-related limitations include laser beam drift^8^, interferometer resolution^5^, and misalignment^7^. Environmental factors and data processing also introduce uncertainties. Sampling strategy^12^, uncertainty evaluation methods^13–15^, and algorithms for error assessment^16,17^ significantly influence result reliability. Statistical approaches such as Monte Carlo simulation^14,15,18^ are widely employed for uncertainty analysis and optimization.

Current trends in straightness metrology emphasize high precision, multi-degree-of-freedom integration, real-time monitoring, and large-scale measurement. Novel techniques including polarization-based interferometry^19^, dual-sensor collaboration^20^, and multi-station time-sharing systems^21^ continue to enhance performance. Applications have expanded from laboratory settings to industrial environments, enabling online geometric error monitoring for H-type drive stages^13^ and long-range reference systems^3^, thereby providing critical support for precision manufacturing. However, when it comes to measuring long tracks with a length greater than 100 m, often limited by numerous challenges such as poor on-site visibility conditions, numerous environmental interference factors, low measurement efficiency, and the difficulty in establishing high-precision linear references, it is hard to meet the demanding accuracy requirements of ± 0.5 millimeters or even higher. Methods like laser alignment require stable, uninterrupted reference lines, which are often impractical on site. While the total station free-station method offers the necessary flexibility, its accuracy is highly sensitive to station placement and instrument error characteristics. There is a lack of a systematic, predictive framework to guide the selection of instrument specifications and station layouts to achieve guaranteed sub-millimeter accuracy in such challenging settings.

To bridge this gap, this study proposes an optimized free-station methodology. The primary contributions are threefold. Firstly, the nonlinear mathematical model for calculating the deviation of the measurement point from the reference line is derived. Then, the Monte Carlo simulation method is adopted to quantitatively analyze the influences of angular measurement error, distance measurement error, and the layout of the total station on the accuracy of the free station method, to determine the instrument selection criteria and the optimization plan for station layout to improve the overall deviation measurement accuracy. Finally, taking the straightness detection of a 160-meter-long track as an example, the proposed optimization strategy is applied to verify the effectiveness of this method in achieving sub-millimeter accuracy measurement in complex environments.

## Principle of deviation measurement with the free station method

**Fig. 1:**
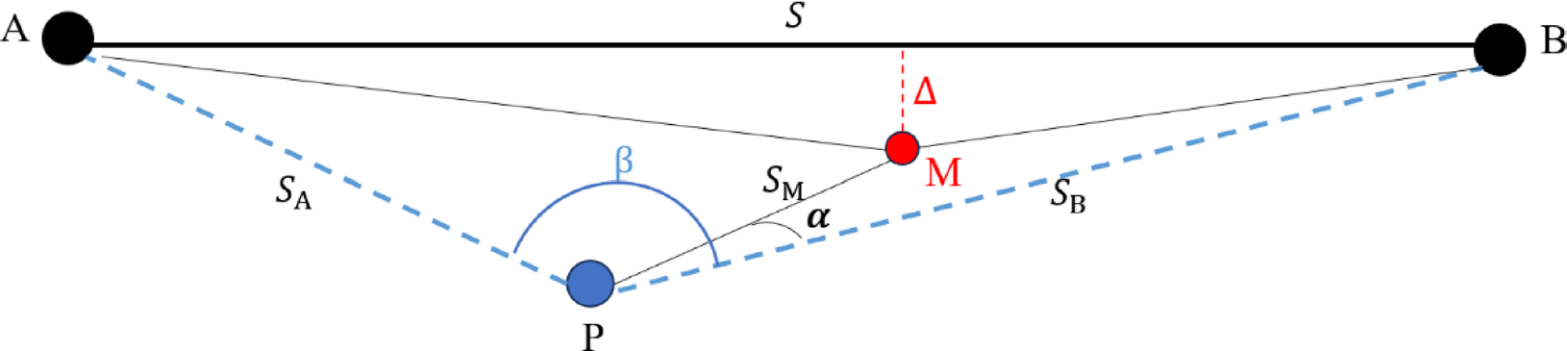
Principle of deviation measurement based on the free station method using a total station.

The free station method for deviation measurement with a total station combines the minor angle method and the large-angle method. This not only makes the installation position of the total station flexible and suitable for on-site obstruction problems, but also ensures the measurement accuracy. As shown in Fig. 1, measurement point M is near the reference line AB. Theoretically, M should lie on the reference line AB. However, due to errors of the measuring instrument and manual operation, M can only approach the reference line AB as closely as possible. And the distance from M to the reference line is called the deviation, marked as $\varDelta$.

In Fig. 1, the total station is set up at point P. Suppose the distance from point P to reference point A is $\:{S}_{A}$, the distance from point P to reference point B is $\:{S}_{B}$, the distance from point P to measurement point M is $\:{S}_{M}$, and the distance between A and B is $\:S$. Assume the horizontal angle reading of the total station aimed at reference point A at point P is $\:{L}_{A}$, and the horizontal angle aimed at point B is $\:{L}_{B}$, and the horizontal angle aimed at measurement point M is $\:{L}_{M}$. Then, the angle APB is $\:\beta\:={L}_{B}-{L}_{A}$, the angle MPB is $\:\alpha\:={L}_{B}-{L}_{M}$, and the angle APM is $\:\beta\:-\alpha\:$.

In Fig. 1, connect measurement point M with points A and B, respectively. Based on the area of the triangle, the area of triangle ABP, $\:{S}_{\varDelta\:\mathrm{A}\mathrm{B}\mathrm{P}}$, is composed of the area of triangle ABM, $\:{S}_{\varDelta\:\mathrm{A}\mathrm{B}\mathrm{M}}$, the area of triangle APM, $\:{S}_{\varDelta\:\mathrm{A}\mathrm{P}\mathrm{M}}$, and the area of triangle PMB, $\:{S}_{\varDelta\:\mathrm{P}\mathrm{M}\mathrm{B}}$. According to the formula for calculating the area of a triangle, $\:{S}_{\varDelta\:\mathrm{A}\mathrm{B}\mathrm{P}}=\frac{1}{2}{S}_{A}{\cdot\:S}_{B}\cdot\:\mathrm{s}\mathrm{i}\mathrm{n}\beta\:$, $\:{S}_{\varDelta\:\mathrm{A}\mathrm{P}\mathrm{M}}=\frac{1}{2}{S}_{A}\cdot\:{S}_{M}\cdot\:\mathrm{s}\mathrm{i}\mathrm{n}\left(\beta\:-\alpha\:\right)$, $\:{S}_{\varDelta\:\mathrm{P}\mathrm{M}\mathrm{B}}=\frac{1}{2}{S}_{B}\cdot\:{S}_{M}\cdot\:\mathrm{s}\mathrm{i}\mathrm{n}\alpha\:$, $\:{S}_{\varDelta\:\mathrm{A}\mathrm{B}\mathrm{M}}=\frac{1}{2}S\cdot\:$. As $\:{S}_{\varDelta\:\mathrm{A}\mathrm{B}\mathrm{P}}={S}_{\varDelta\:\mathrm{A}\mathrm{P}\mathrm{M}}+{S}_{\varDelta\:\mathrm{P}\mathrm{M}\mathrm{B}}+{S}_{\varDelta\:\mathrm{A}\mathrm{B}\mathrm{M}}$, based on the principle of triangle area calculation, it can be written as:

1 $$\:\frac{1}{2}{S}_{A}{\cdot\:S}_{B}\cdot\:\mathrm{s}\mathrm{i}\mathrm{n}\beta\:=\frac{1}{2}{S}_{A}\cdot\:{S}_{M}\cdot\:\mathrm{s}\mathrm{i}\mathrm{n}\left(\beta\:-\alpha\:\right)+\frac{1}{2}{S}_{B}\cdot\:{S}_{M}\cdot\:\mathrm{s}\mathrm{i}\mathrm{n}\alpha\:+\frac{1}{2}S\cdot\:$$

(1).

Then the expression of the deviation $\varDelta$ from measurement point M to the reference line AB is:

2 $$\varDelta {\mathrm{=}}\frac{{{S_A} \cdot {S_B} \cdot \sin \beta - {S_B} \cdot {S_M} \cdot \sin \alpha - {S_A} \cdot {S_M} \cdot \sin \left( {\beta - \alpha } \right)}}{S}$$

In Fig. 1, the measurement point M is below the reference line AB, the value of the deviation $\varDelta$ is positive. If the measurement point M is above the reference line AB, the value of the deviation $\varDelta$ will be negative because the sum of $\:{S}_{\varDelta\:\mathrm{P}\mathrm{M}\mathrm{B}}$ and $\:{S}_{\varDelta\:\mathrm{P}\mathrm{M}\mathrm{B}}$ is larger than $\:{S}_{\varDelta\:\mathrm{A}\mathrm{B}\mathrm{P}}$. In practice, measurement point M is judged to be above reference line AB if $\varDelta$ < 0, and below if $\varDelta$ > 0.

## Deviation accuracy analysis of the free station method

### Accuracy analysis of the free station method using Monte Carlo simulation

**Fig. 2:**
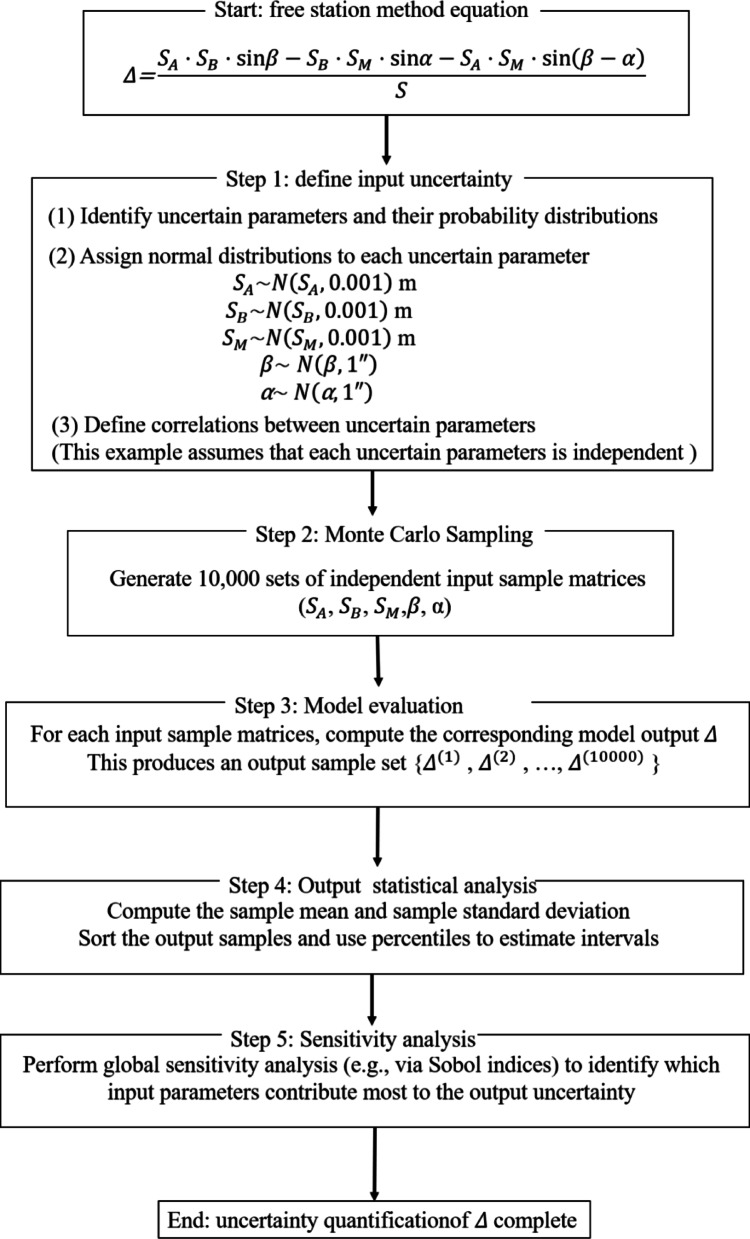
Accuracy analysis of deviation using Monte Carlo simulation method.

As Eq. (2) is a nonlinear expression and involves multiple variables, taking the derivative of this equation will become extremely complicated. The Eq. (2) has two kinds of observations including angle observation and distance observation. To quantitatively analyze the measurement uncertainty of the free station method, the Monte Carlo simulation method is adopted to conduct uncertainty propagation analysis on the nonlinear Eq. (2). Here, the accuracy analysis of free station method is shown in Fig. 2.

In Fig. 2, assuming that both the angle measurement error and the distance measurement error follow a normal distribution, where the standard deviation of the angle measurement is 1 arcsecond and the standard deviation of the distance measurement is 1 mm. 10,000 sets of input quantity samples are generated through Latin hypercube sampling, and the statistical characteristics of the output quantity are calculated after model propagation. The standard deviation of the samples is obtained based on the Bessel formula as the synthetic standard uncertainty, and the normality of the output is verified through kernel density estimation. During the simulation process, the Sobol sequence is adopted to enhance the sampling efficiency and ensure that the Monte Carlo standard error is less than 1% of the total uncertainty. The final result provides an extended uncertainty with a probability of 95%, fully reflecting the nonlinear characteristics of the measurement model and the interaction between the input quantities.

### Contribution of angle measurement and distance measurement accuracy to the deviation accuracy

**Fig. 3:**
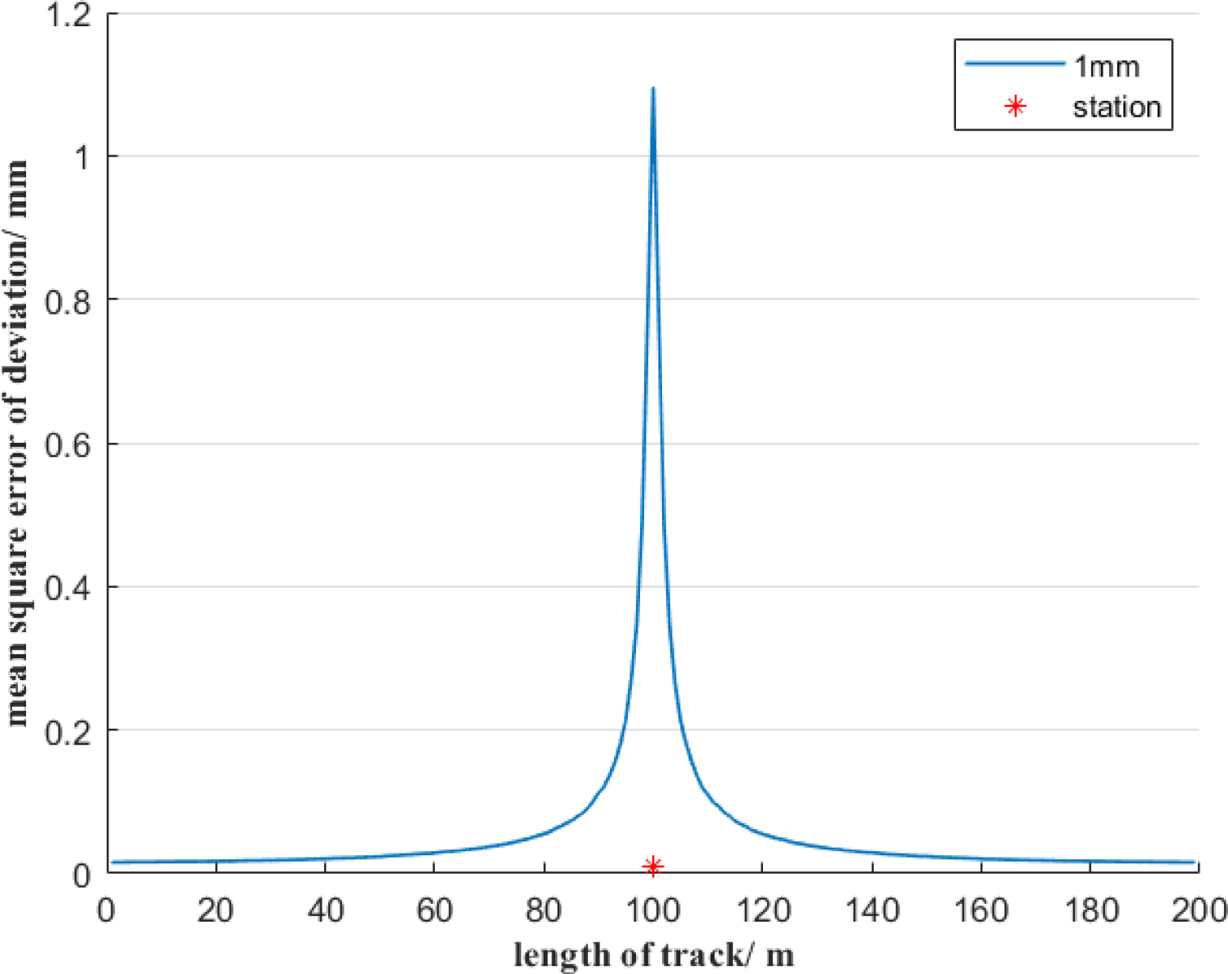
Uncertainty of deviation of the free station method when only considering the distance measurement error.

**Fig. 4:**
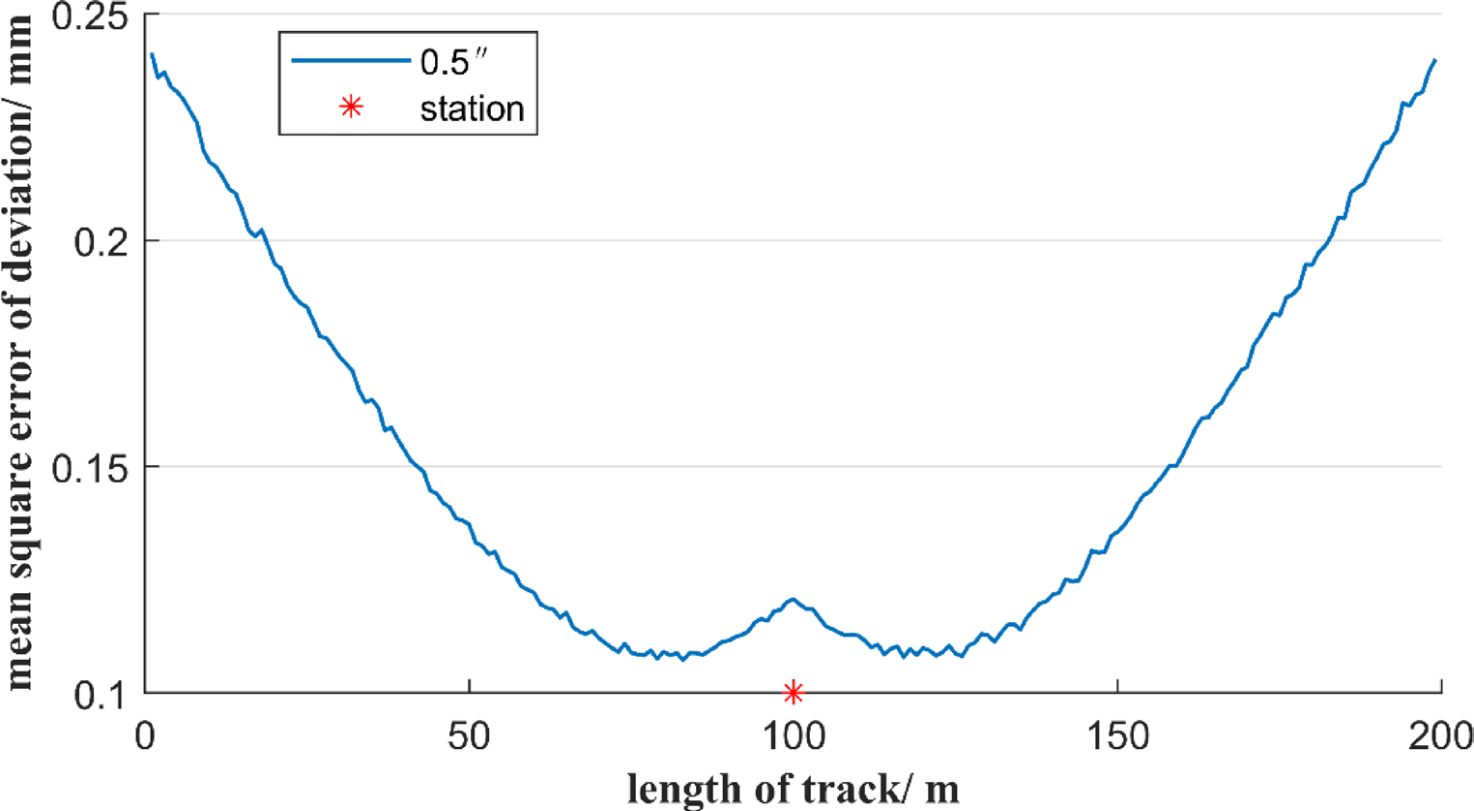
Uncertainty of deviation of the free station method when only considering angle measurement error.

In Fig. 1, if the distance of reference line AB is 200 m, and the total station is set at the center of the reference line AB and is 1 m away from it, then the uncertainty of the deviation of the free station method will be estimated using Monte Carlo simulation method. To analyze the contribution of angle measurement error and distance measurement error on the free station method respectively, it is divided into the following two situations. Firstly, assuming error-free angle measurements, we analyze the mean square error of deviation induced solely by distance measurement errors in the free station method, as illustrated in Fig. 3. Secondly, assuming perfect distance measurements, we quantify how angle measurement errors alone affect the mean square error of deviation using the free station method, as demonstrated in Fig. 4.

As can be seen from Fig. 3, when only the ranging error is considered, the mean square error of the deviation of the deviation using the free station method is symmetrically distributed with regard to the position of the total station. The mean square error of the deviation of measurement points from the reference point to the station first increases slowly and then increases sharply. When the measurement point is within a range of 20 m from the total station, the mean square error of deviation of the measurement points changes sharply, with the value varying from 0.01 mm to 1.1 mm, and the maximum value is 18 times the minimum value.

**Fig. 5:**
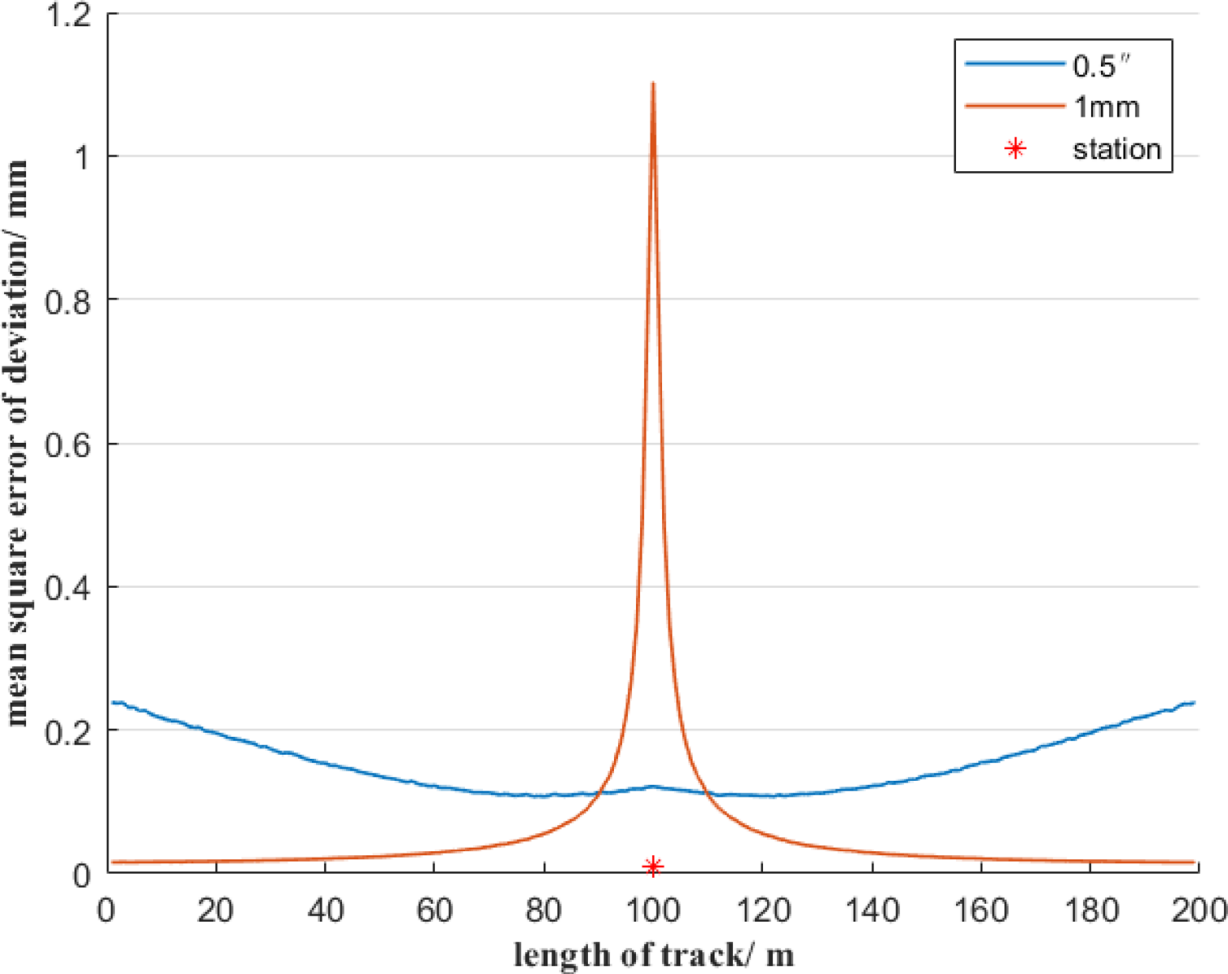
Comparison of the influences of angle measurement and distance measurement errors on the uncertainty of deviation by the free station method.

As shown in Fig. 4, when only the angle measurement error is considered, the mean square error of deviation of the measurement points using the free station method is symmetrically distributed concerning the position of the total station. The mean square error of the deviation of measurement points from the reference point to the total station first decreases and then increases. When the measurement point is within a 20 m range from the total station, the mean square error of the deviation of the measurement point shows an increasing trend, with the value changing from 0.11 mm to 0.12 mm. The mean square error of the deviation of the other measurement points decreases slowly, with the value decreasing from 0.25 mm to 0.11 mm. The maximum value is about 2 times the minimum value. To further compare the influence of distance measurement error and angle measurement error factors on the uncertainty of deviation using the free station method, Figs. 3 and 4 are combined as shown in Fig. 5.

In Fig. 5, the mean square error of deviation of the measurement points shows a symmetrical distribution with regard to the position of the total station. When the distance between a measurement point and the total station is less than 10 m, the ranging error of the total station has a dominant influence on the mean square error of deviation. When the distance between the measurement point and the total station is within the range of 10 to 100 m, the angle measurement error has a significant impact on the mean square error of deviation. Therefore, when the measurement point is far from the total station, a high-precision angle-measuring total station should be considered for selection. However, when the measurement point is short from the total station, the distance measurement accuracy must be guaranteed simultaneously. In particular, when the distance between the measuring point and the total station is within 10 m, the mean square error of deviation of the measurement point is relatively large, which basically cannot meet the measurement requirement of a deviation accuracy of ± 0.5 mm.

**Fig. 6:**
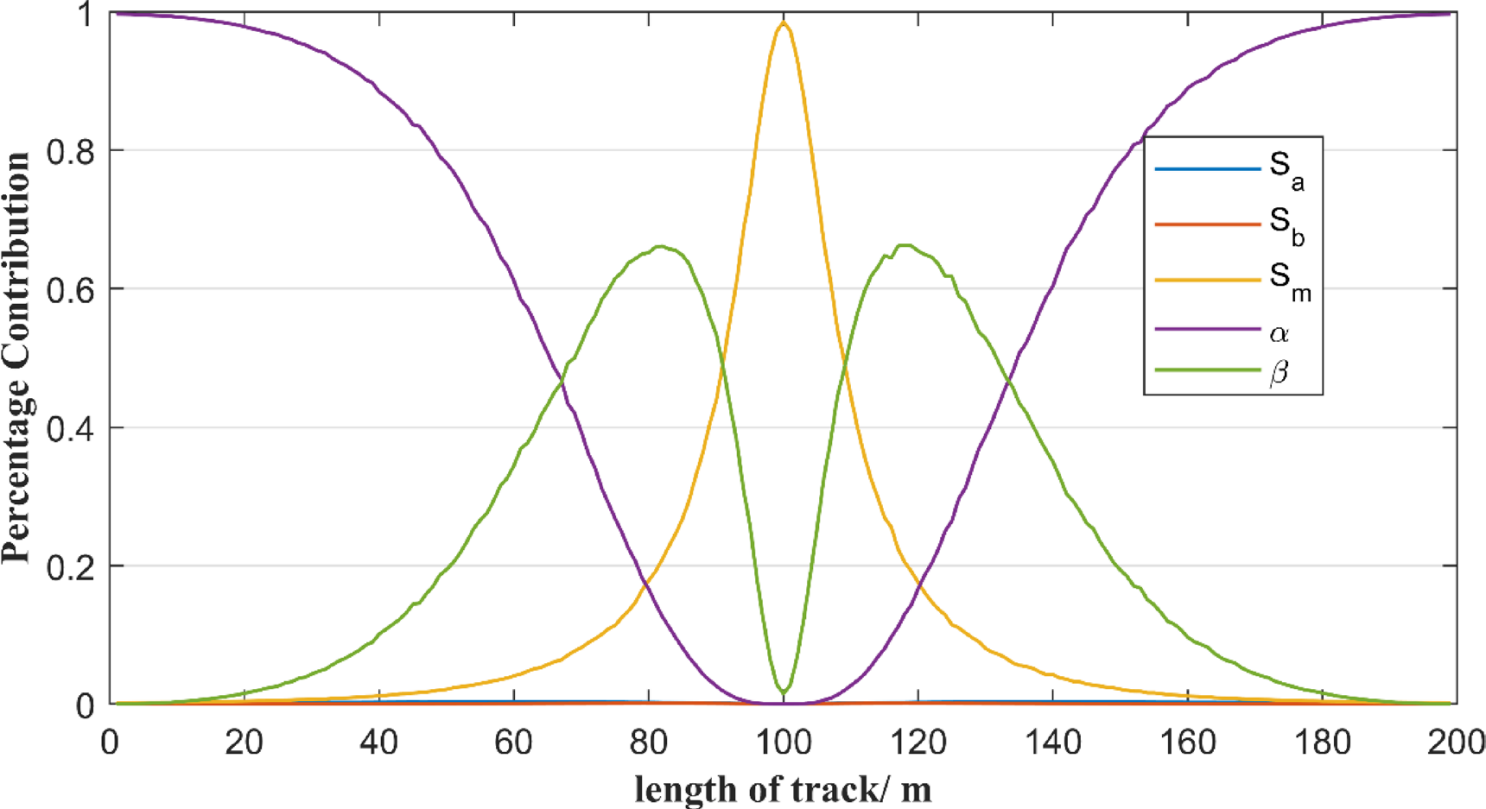
Contribution of each parameter to the free station method.

To quantify parameter influences more precisely, the contributions of Eq. (2) parameters to free station method accuracy are refined by using Sobel indices. The total station is set up in the middle of the reference line AB and is 1 m away from the reference line. In Eq. (2), parameters include $\:{S}_{A}$, $\:{S}_{B}$, $\:{S}_{M}$, $\:\alpha\:$ and $\:\beta\:$. To perform the analysis of each parameter contribute to the uncertainty of the output, the percentage contribution of each parameter calculated by Sobel indices is shown in Fig. 6.

As shown in Fig. 6, on this 200-meter reference line AB, the parameter indicating the maximum contribution of each point on the reference line to the result varies with the distance from the total station. When the measurement points are near the ends of the reference line and the distance from the total station is greater than 65 m, the contribution of parameter $\:\alpha\:$ to the uncertainty of the output is the greatest. When the measurement points are near the middle of the reference line and the distance from the total station is less than 10 m, the contribution of parameter $\:{S}_{M}$ to the uncertainty of the output is the greatest. When the distance from measurement point to the total station is between 10 m and 65 m, the contribution of parameter $\:\beta\:$ to the uncertainty of the output is the greatest. Besides, the contributions of parameters $\:{S}_{A}$ and $\:{S}_{B}$ to the uncertainty of the output can be ignored.

**Table 1:** Parameters’ contributions of each point on the reference line to the output.

Point on the reference line (m)	Contribution of parameters to output (%)				
	$\:{S}_{A}$	$\:{S}_{B}$	$\:{S}_{M}$	$\:\alpha\:$	$\:\beta\:$
1	0.17	0.00	0.17	99.66	0.00
65	0.33	0.00	5.79	50.66	43.22
80	0.25	0.11	17.86	16.60	65.18
100	0.00	0.00	98.54	0.00	1.46
120	0.26	0.12	17.53	16.69	65.40
135	0.35	0.00	5.57	50.68	43.40
199	0.18	0.00	0.17	99.65	0.00

In Table 1, if the measurement point is on the ends of the reference line AB, such as the location of 1–199 m, the percentage contribution of $\:\alpha\:$ to the output is 99.66% or 99.65%. The other parameters’ percentage contribution to the output is negligible. When the measurement point is located at the midpoint of reference line AB, specifically at 100 m, the percentage contribution of $\:{S}_{M}$ to the output is 98.54%, while that of $\:\beta\:$ accounts for 1.46%. Contributions from other parameters to the output are negligible. When the measuring point is 80–120 m in the reference line, the percentage contribution of $\:\beta\:$ to the output is about 65%, while that of $\:{S}_{M}$ accounts for 17% and that of $\:\alpha\:$ accounts for 16%.Combining Fig. 6; Table 1, it can be seen that the contribution of parameter $\:\alpha\:$ to the output decreases as the distance between the measurement point and the total station decreases, while the contribution of parameter $\:{S}_{M}$ to the output increases as the distance between the measurement point and the total station decreases. The contribution of parameter $\:\beta\:$ to the output first gradually increases and then sharply decreases as the distance between the measurement point and the total station decreases. In conclusion, while measurement point is near total station, the contribution of $\:{S}_{M}$ to the output is largest. While measurement point is far from total station, the contribution of $\:\alpha\:$ to the output is largest.

### Influence of different total station measurement accuracies on deviation accuracy

**Fig. 7:**
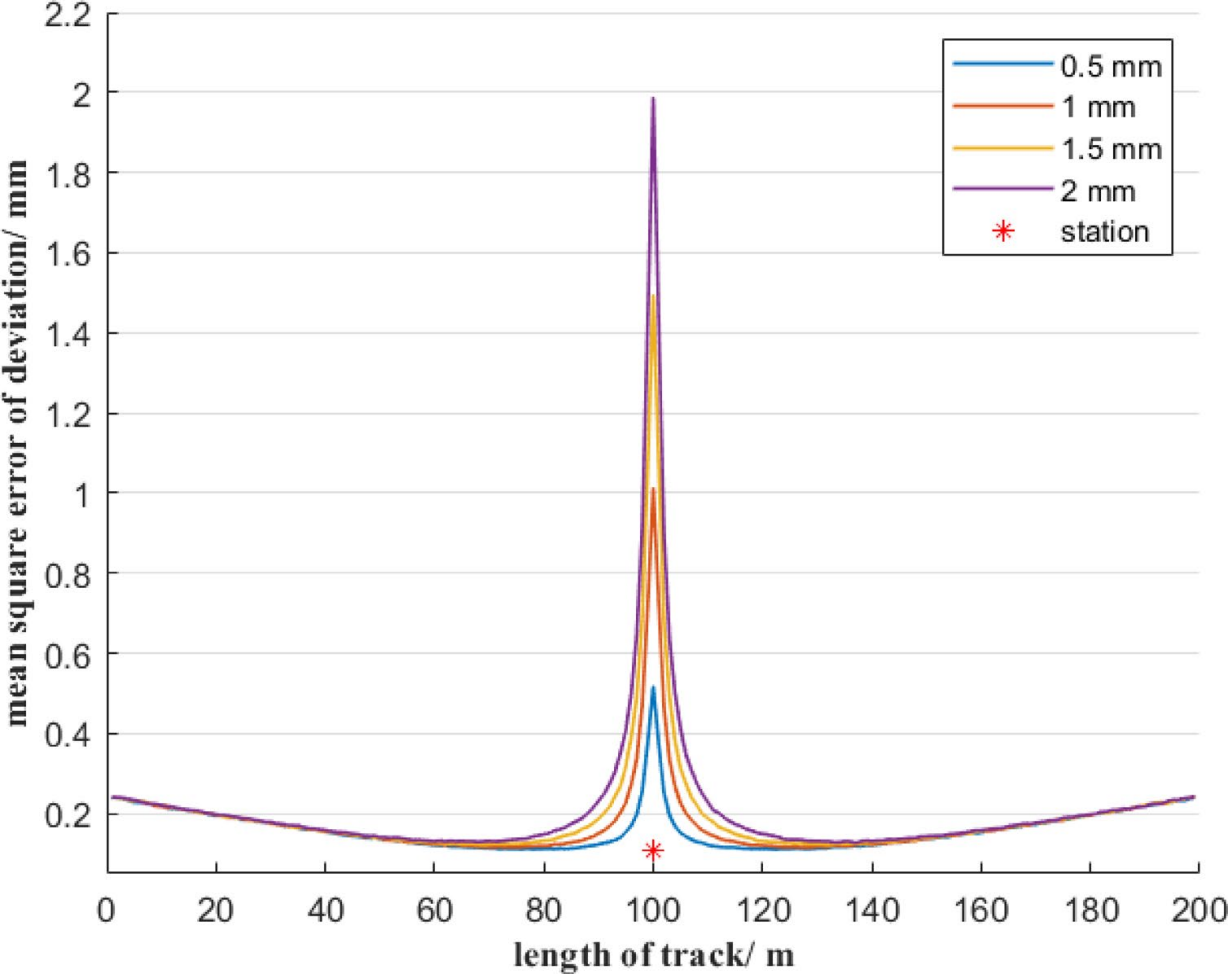
Effect of different ranging errors on the accuracy of the deviation of the free station method.

**Fig. 8:**
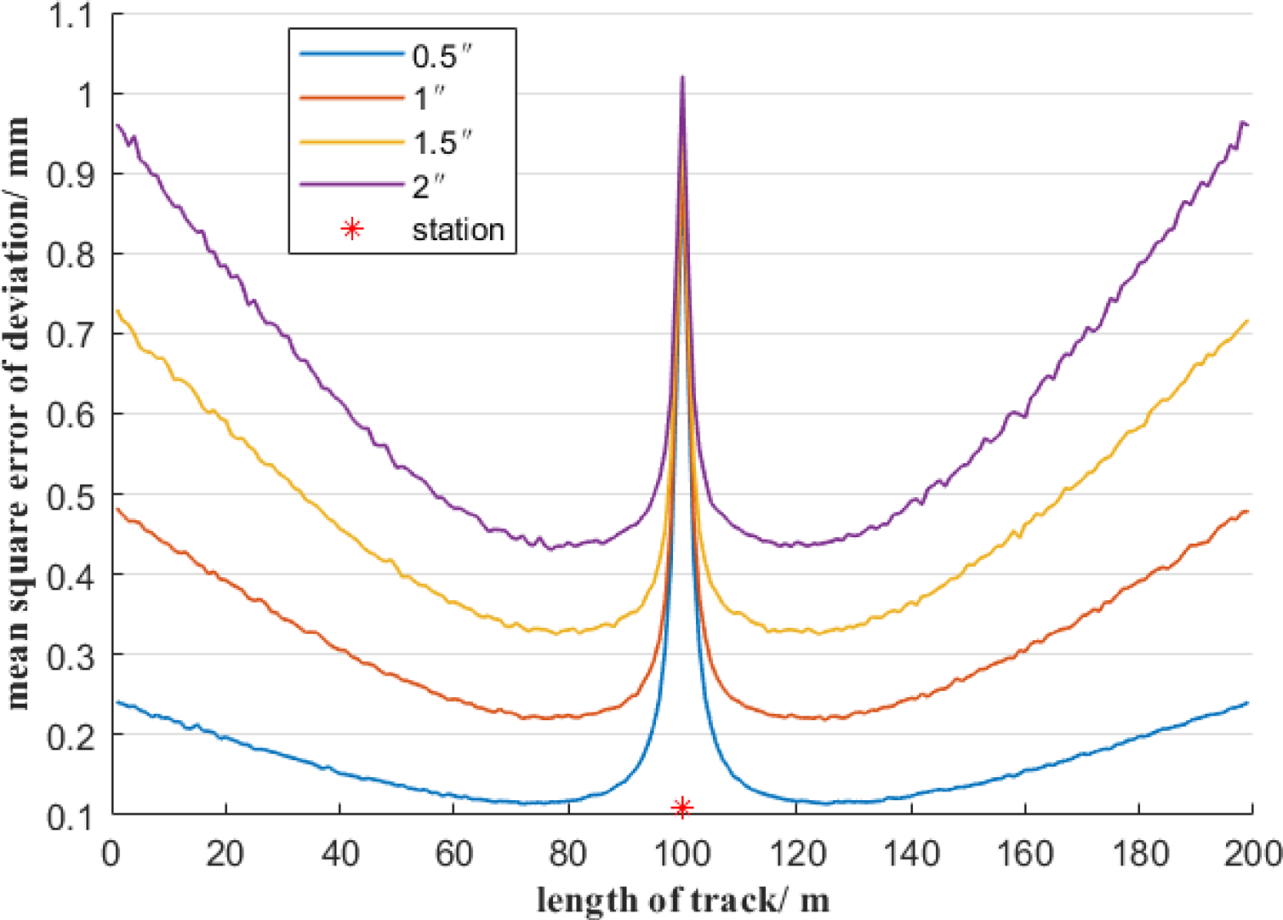
Effect of different angle errors on the accuracy of the deviation of the free station method.

Furthermore, it is analyzed the influence of total stations with different measurement accuracies on the mean square error of deviation. In the first case, the total station’s angular measurement accuracy is 0.5″, and the total station is set up in the middle of a 200-meter reference line AB and is 1 m away from it. When the total station’s distance measurement accuracy is 0.5 mm, 1 mm, 1.5 mm, and 2 mm, respectively, the distribution of the mean square error of deviation at each measurement point on the reference line AB, based on the free station method, is shown in Fig. 7. In the second case, assume the total station’s distance measurement accuracy is 1 mm, and the total station is set up in the middle of a 200-meter reference line AB and is 1 m away from it. When the total station’s angular measurement accuracy is 0.5″, 1″, 1.5″, and 2″, respectively, the distribution of the mean square error of deviation at each measurement point on the reference line AB is shown in Fig. 8.

As can be seen from Fig. 7, although the distance measurement accuracy of total stations varies, the distribution trend of the mean square error of the measurement point deviation is consistent. At the position where the total station is located, the mean square error of the measurement point deviation reaches its maximum value, and this maximum value is close to the distance measurement accuracy of the total station. Besides, the mean square error of the deviation of the measurement points is symmetrically distributed at the position of the total station. As the distance between the measurement point and the total station increases, the mean square error of the deviation of the measurement points first decreases rapidly and then increases slowly. When the measurement point is 20 to 100 m away from the total station, although the distance measurement accuracy of the total station varies, the mean square error value of the measurement point deviation is the same. However, within a range of 20 m between the measurement point and the total station, the mean square error of the measurement point deviation increases significantly as the distance measurement accuracy decreases. Figure 7 shows that the mean square error of deviation of the measurement points near the total station is mainly positively correlated with the ranging error.

As shown in Fig. 8, though total stations with different angular measurement accuracies are selected, the mean square error distribution of the measurement point deviation still shows a symmetrical distribution about the position of the total station, and the mean square error of the measurement point deviation reaches the maximum value at the total station position. As the distance between the measurement point and the total station increases, the mean square error of the measurement point deviation first decreases sharply and then gradually increases. When the angle measurement accuracy decreases from 0.5″ to 2″, the mean square error of the measurement point deviation at the starting and ending measurement points of the reference line AB increases significantly. Figure 8 shows that the mean error of the deviation from the measurement points far from the total station is mainly positively correlated with the angular measurement error.

Based on Figs. 7 and 8, it can be known that when using the free station method, a total station with high-precision angular measurement should be selected. When the measurement point is relatively close to the total station, the distance measurement accuracy is the main factor of the mean square error of the deviation of the measurement point. It is necessary to avoid observing measurement points within a distance of 20 m from the total station. When the measurement point is more than 20 m away from the total station, the distance measurement error of the total station has a relatively small impact on the mean square error of deviation of the measurement point. That is, the total station must be selected with an angular measurement accuracy of 0.5″, but the distance measurement accuracy can be within 2 mm.

### Influence of different installation positions of total stations on deviation accuracy

**Fig. 9:**
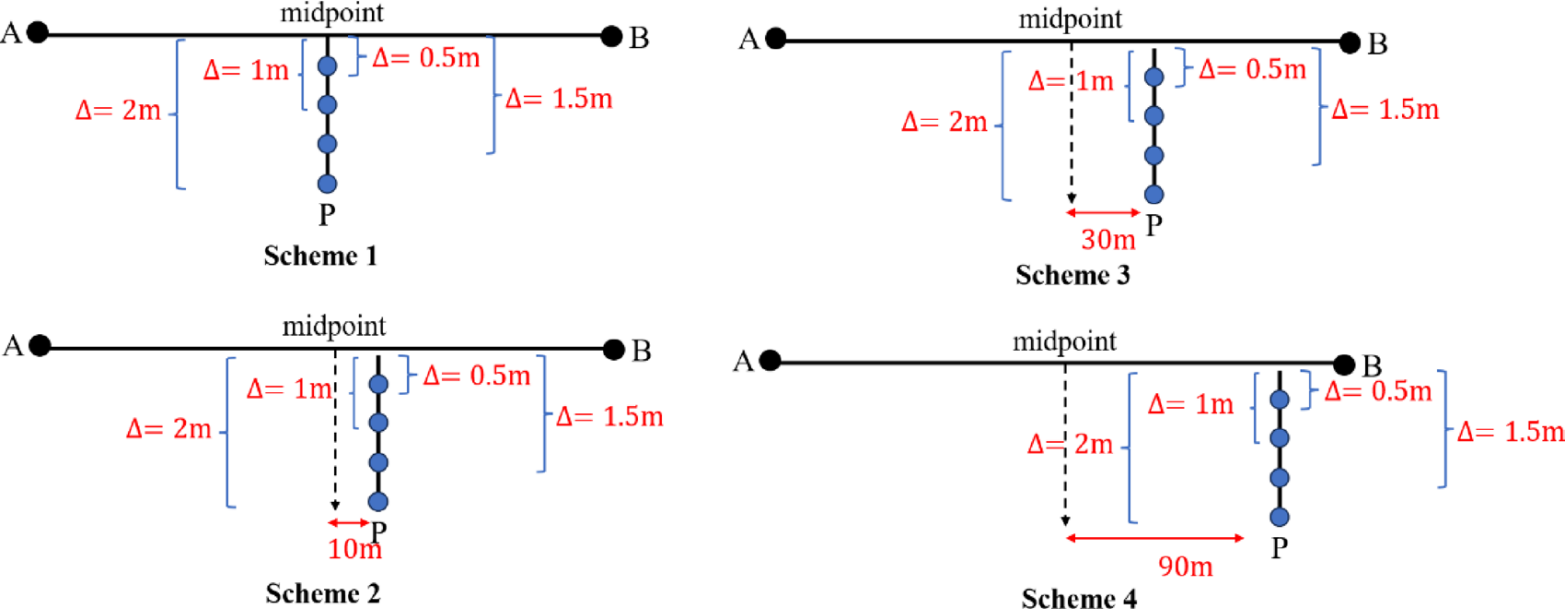
Different station locations for total stations.

 To systematically quantify the influence of total station positions on the deviation accuracy using the free station method, four measurement schemes, shown in Fig. 9, are designed based on the instrument’s relative position to baseline AB whose length is 200 m:

Scheme 1: The total station is positioned along the perpendicular bisector of AB, with orthogonal distances of 0.5 m, 1 m, 1.5 m, and 2 m from the baseline.

Scheme 2: The instrument is offset 10 m from the perpendicular bisector while maintaining the same orthogonal distances (0.5 to 2 m) to AB.

Scheme 3: The offset distance is increased to 30 m from the bisector, preserving the orthogonal distance variations.

Scheme 4: The total station is placed near baseline endpoints with identical orthogonal distance settings

**Fig. 10:**
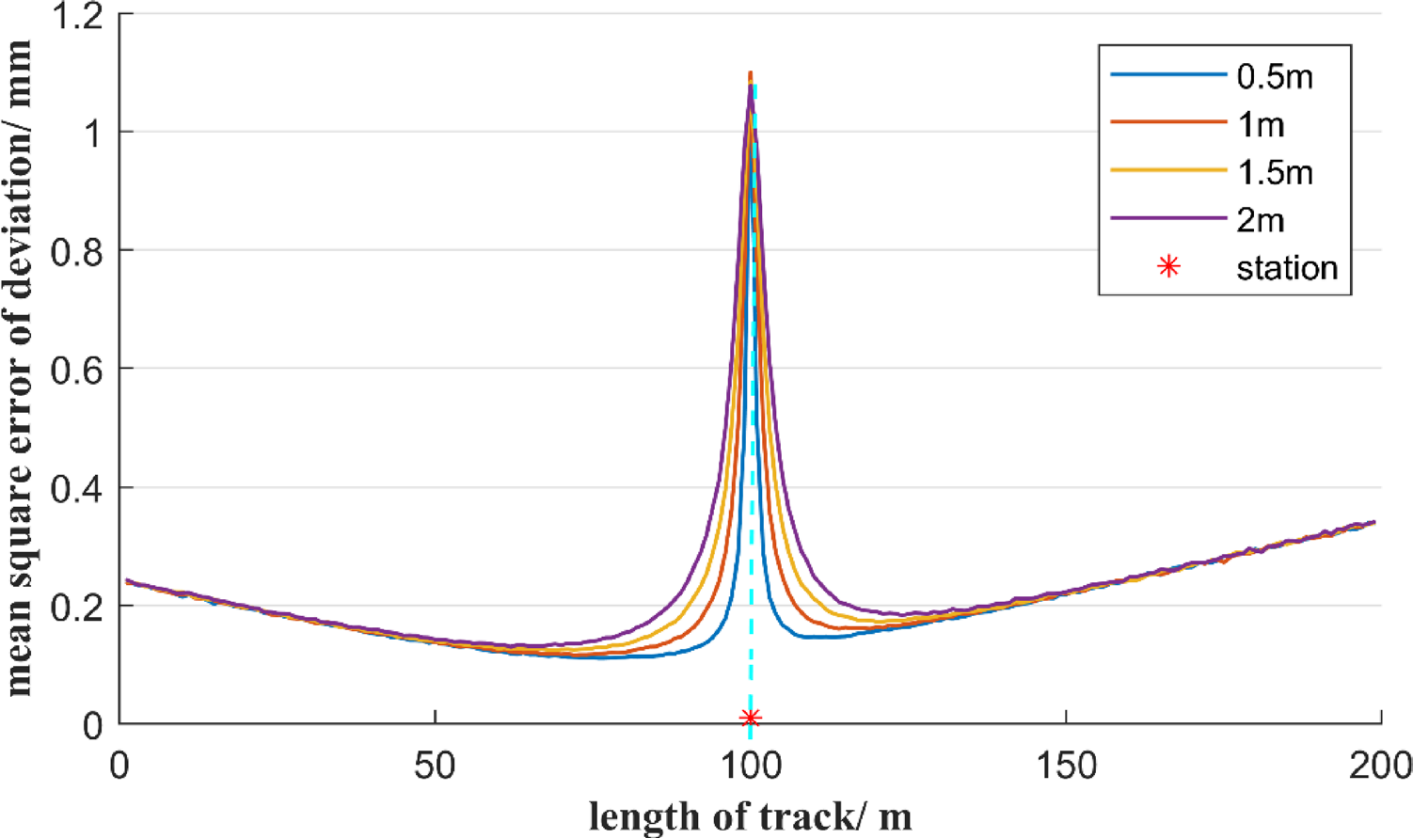
Distribution of the mean square error of the measurement point deviation in scheme 1.

**Fig. 11:**
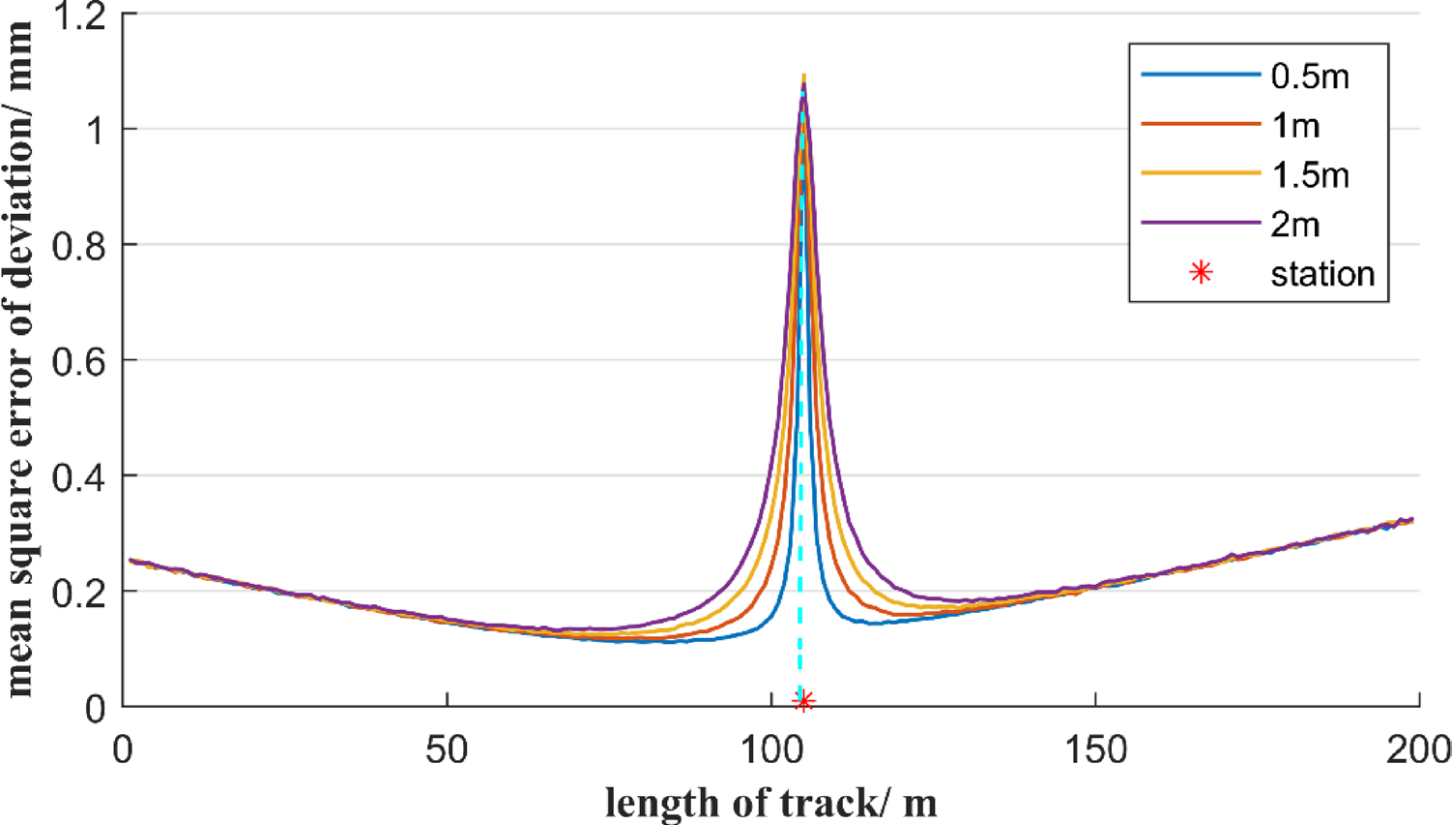
Distribution of mean square error of the measurement point deviation in scheme 2.

**Fig. 12:**
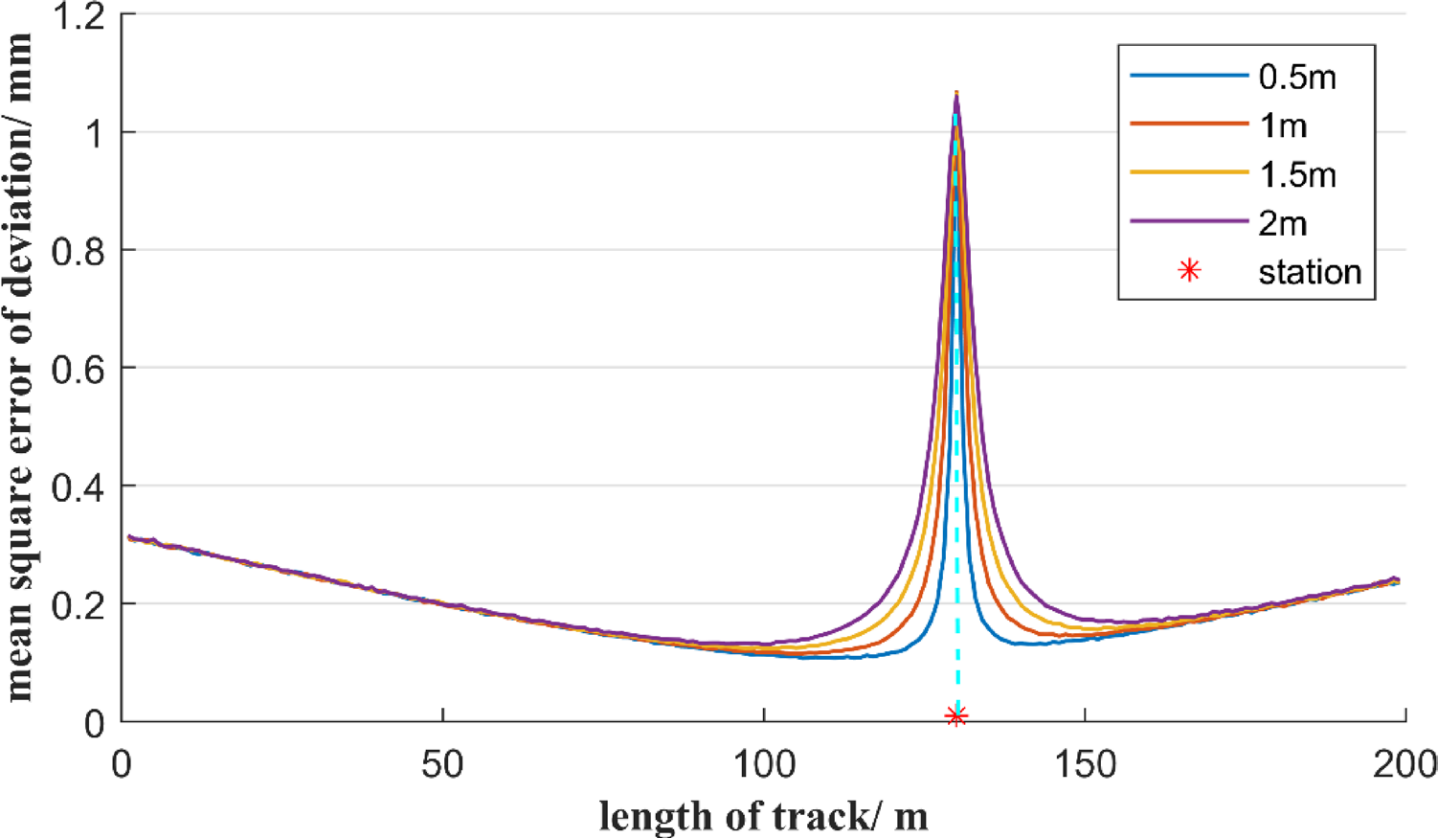
Distribution of mean square error of the measurement point deviation in scheme 3.

**Fig. 13:**
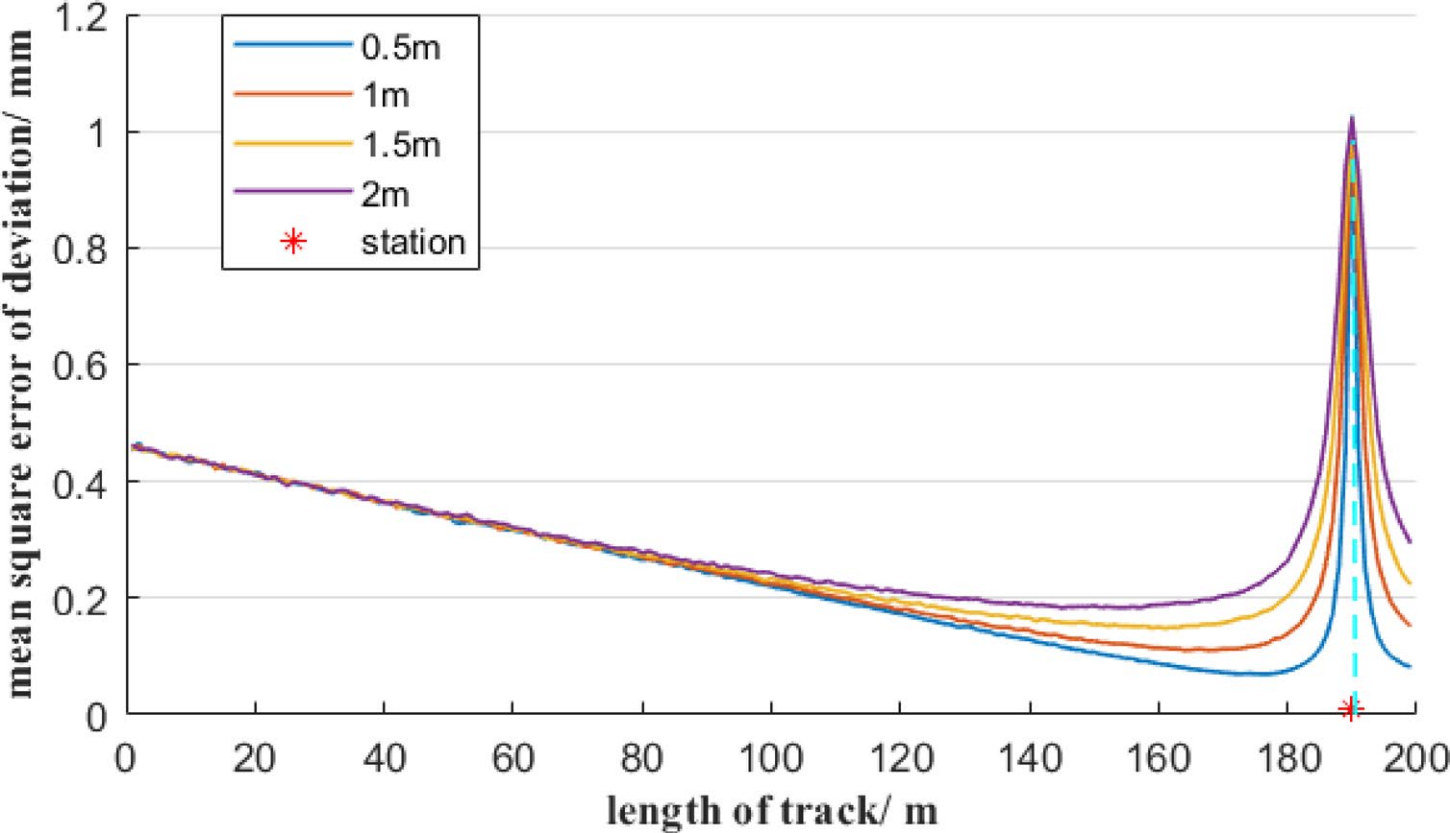
Distribution of the mean square error of the measurement point deviation in scheme 4.

Assume that the angular measurement accuracy of the total station is 0.5″, and the distance measurement accuracy is 1 mm. Based on the principle of Monte Carlo simulation, the mean square error distribution of the deviation in the above four schemes is shown in Figs. 10, 11, 12 and 13.

Based on the simulation results from Figs. 10, 11, 12 and 13, the following conclusion can be drawn:

Despite the different positions of the total stations on the reference line AB, the mean square error distribution of the measurement point deviations all exhibit a symmetrical characteristic centered on the instrument position, and the maximum value always occurs at the location of the total station. In terms of spatial distribution, the mean square error of the measurement points close to the total station shows a sharp upward trend, while the mean square error values of the measurement points far from the instrument are relatively small. Overall, as the distance between the measurement point and the total station increases, the mean square error shows a three-stage change characteristic of first rapidly decreasing, then tending to level off, and finally rising sharply again. Thus, by adjusting the layout of the total station, the mean square error of the deviation distribution of the measurement points along the entire reference line can be effectively optimized. When the vertical distance between the total station and the reference line increases, the range affected by the sharp increase in mean square error expands from 10 m to 20 m. In addition, the reduction of the vertical distance can significantly lower the minimum value of the mean square error at the measurement points, indicating that shortening the distance between the total station and the reference line helps to improve the overall measurement accuracy.

## Application of the free station method in track straightness detection

The track of a certain drag water pool is approximately 160 m long and has a span of 7.6 m. After the foundation construction is completed, the track is initially installed, and the deformation has basically stabilized, it is necessary to adjust its straightness. The straightness throughout the entire range should not exceed ± 0.5 mm. To ensure that the mean square error of each measurement point deviation is less than 0.5 mm, considering influences of measurement environment and manual operation, the mean square error of each measurement point deviation is set at ± 0.2 millimeters. Since the mean square error of the measurement point deviation near the station is close to the mean square error of the distance measurement of the total station, it is necessary to set the total station at different positions on the track to optimize the mean square error value of the measurement point deviation.

**Fig. 14:**
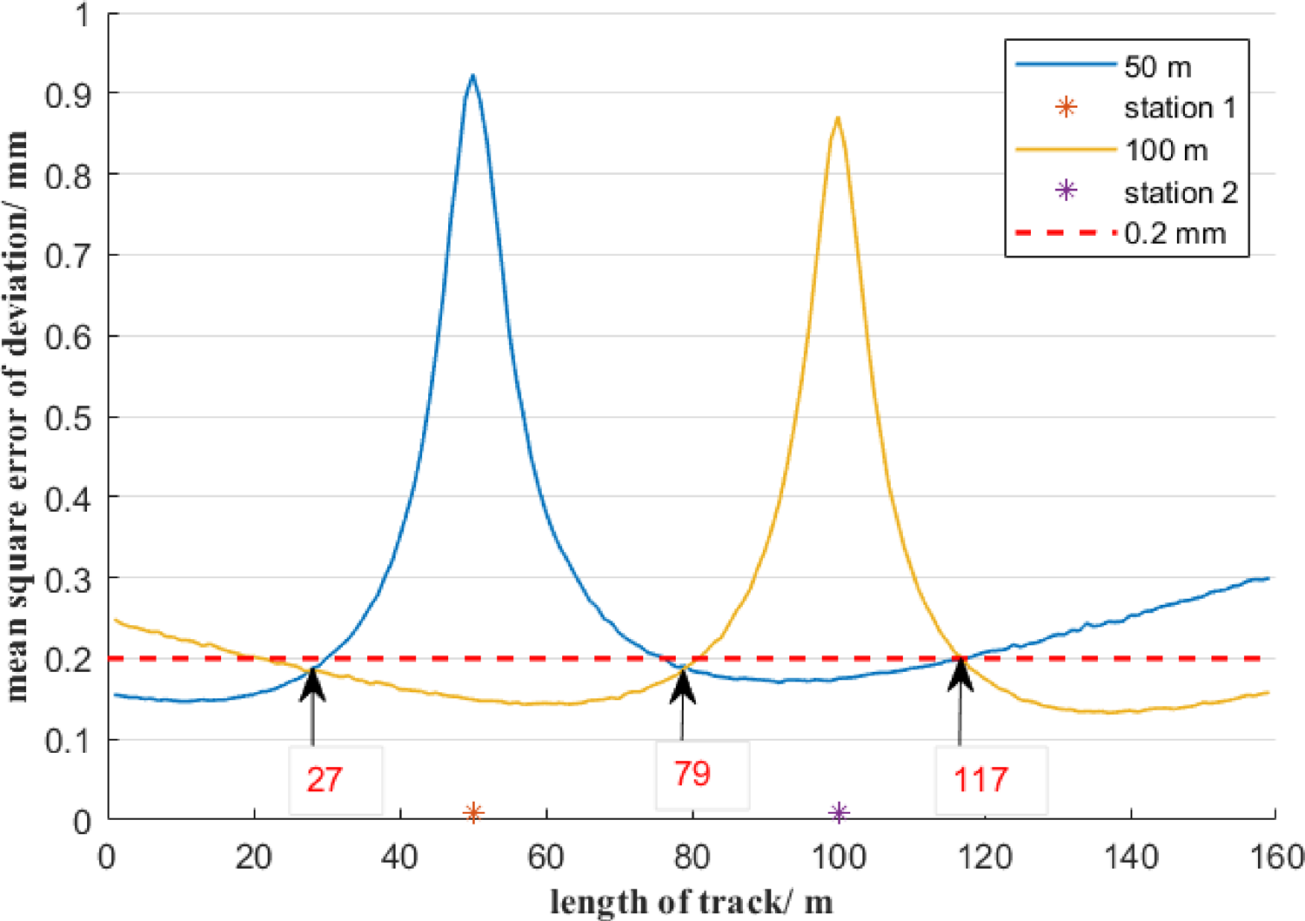
Distribution of the mean square error of the deviation of measurement points by optimal total station location.

The total station is 4 m away from the centerline of the track, to determine the optimal layout of total station, the mean square error distribution of the deviations at each measurement point on the 160-meter-long track is estimated by Monte Carlo simulation method and the estimated result is shown in Fig. 14. Take the center of the track as the reference line, the total station shall be set up at 50 m and 100 m respectively. Figure 14 also shows that when the total station is at 50 m on the track, the mean square error of the deviation at the measurement points 0 to 27 m and 79 to 117 m on the track is less than 0.2 millimeters. When the total station is at 100 m on the track, the mean square error of the deviation at the measurement points 117 to 160 m on the track is less than 0.2 millimeters.

**Fig. 15:**
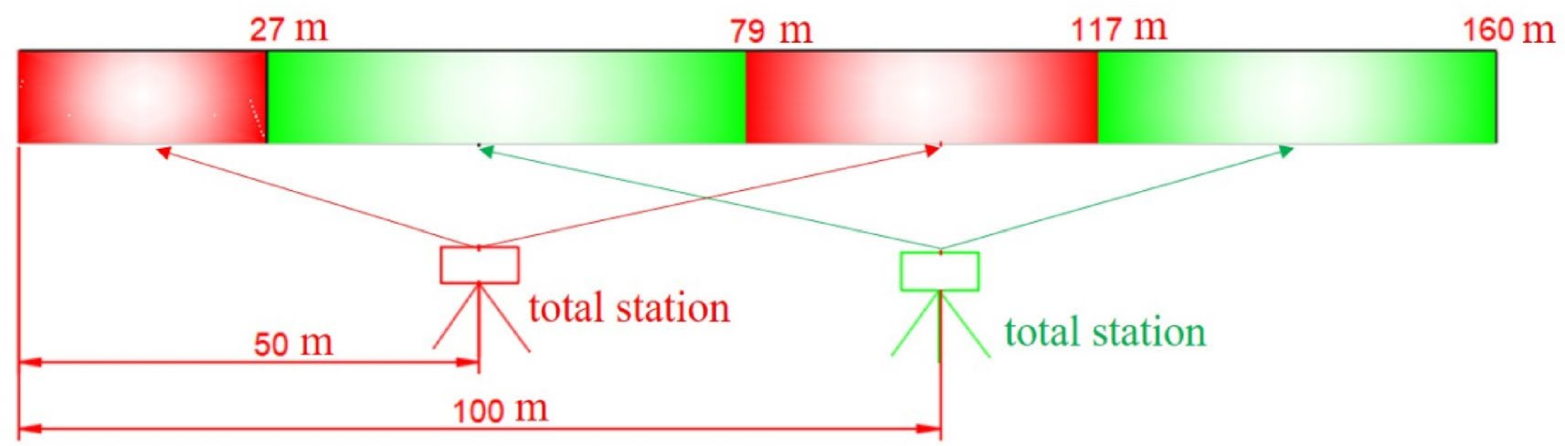
Layout of total station and observation range of total station.

Based on the estimated result, in practice, the total station setting is shown in Fig. 15. Firstly, the total station is set up 50 m away taking the reference of track. And the total station only observes the measurement points at 0 to 27 m and 79 to 117 m on the track. Then, the total station is set up 100 m away taking the reference of track. And the total station only observes the measurement points at 27 to 79 m and 117 to 160 m on the track. The 160 m long track is instrumented with measurement points at 3 m intervals, starting at the initial position (0 m). This arrangement produced 54 measurement locations, with the final point at 159 m.

**Fig. 16:**
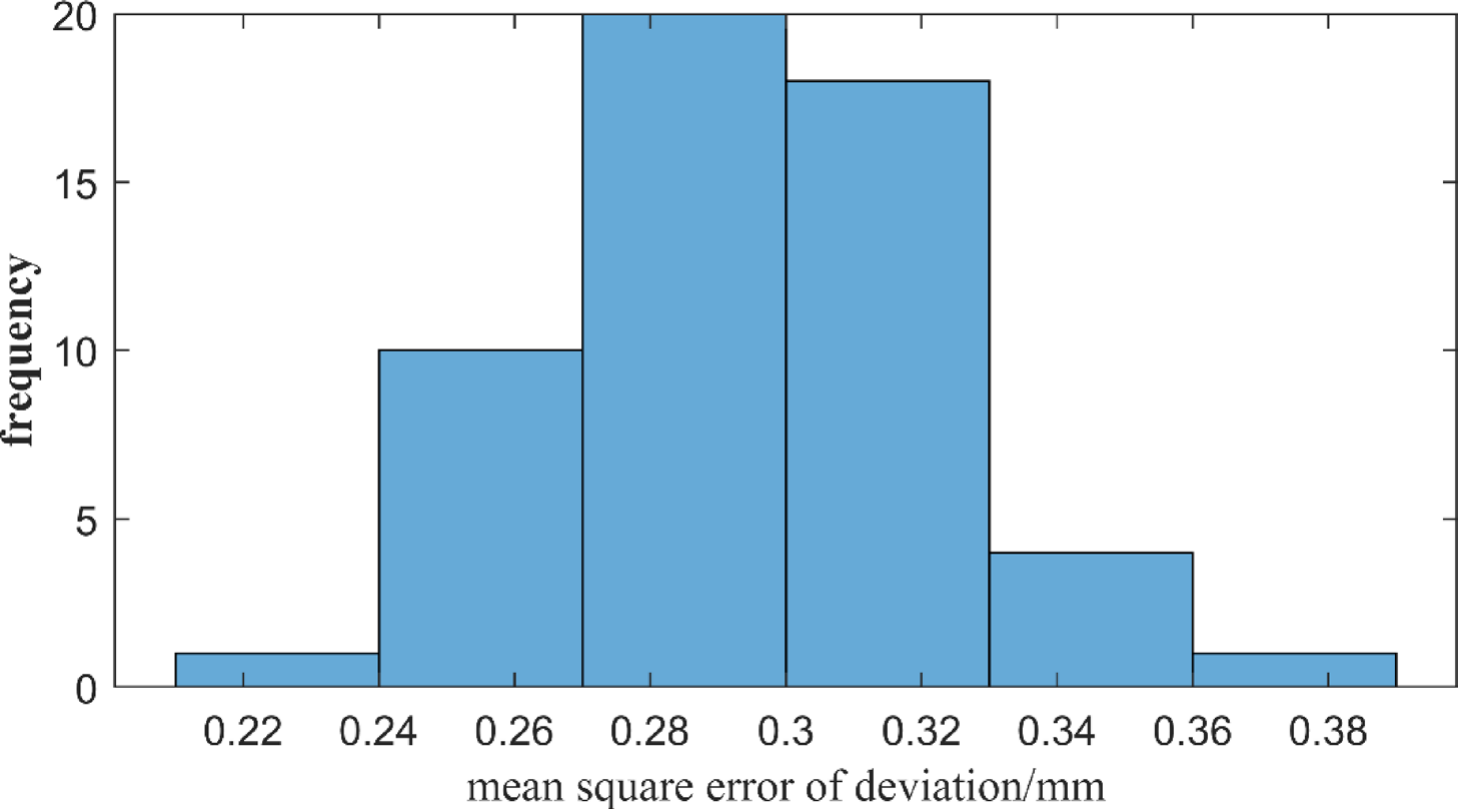
Frequency distribution of the mean square error of the deviation of all measurement points in alignment.

To evaluate the accuracy of the measurement results and considering the influence of environmental factors on the measurement results, under the condition that the installation position of the total station and the measurement range of the total station are fixed, deviation of each point is repeatedly measured at different times over three days, totaling 6 times. It is held respectively from 9:00 a.m. to 11:00 a.m., from 3:00 p.m. to 5:00 p.m. Therefore, there are six deviation values at each point in term of the reference. According to the Bessel’s formula, the mean square error of deviation at each point is obtained. The frequency distribution of the mean square errors of all points is shown in the Fig. 16.

As can be seen from Fig. 16, the mean square error of the measurement point deviation is concentrated in the range of 0.27 mm to 0.33 mm, accounting for approximately 71%. The mean square error ranging from 0.24 mm to 0.27 mm accounts for approximately 18%. The mean square error of the measurement point deviation greater than 0.33 mm accounts for approximately 9%. The mean error of the measurement point deviation less than 0.24 mm accounts for approximately 2%. From this, it can be known that the mean square error of the straightness measurement result at 160 m is approximately ± 0.30 mm, but this result is larger than the theoretical analysis mean square error of 0.2 mm which might be caused by the various on-site measurement environment.

**Fig. 17:**
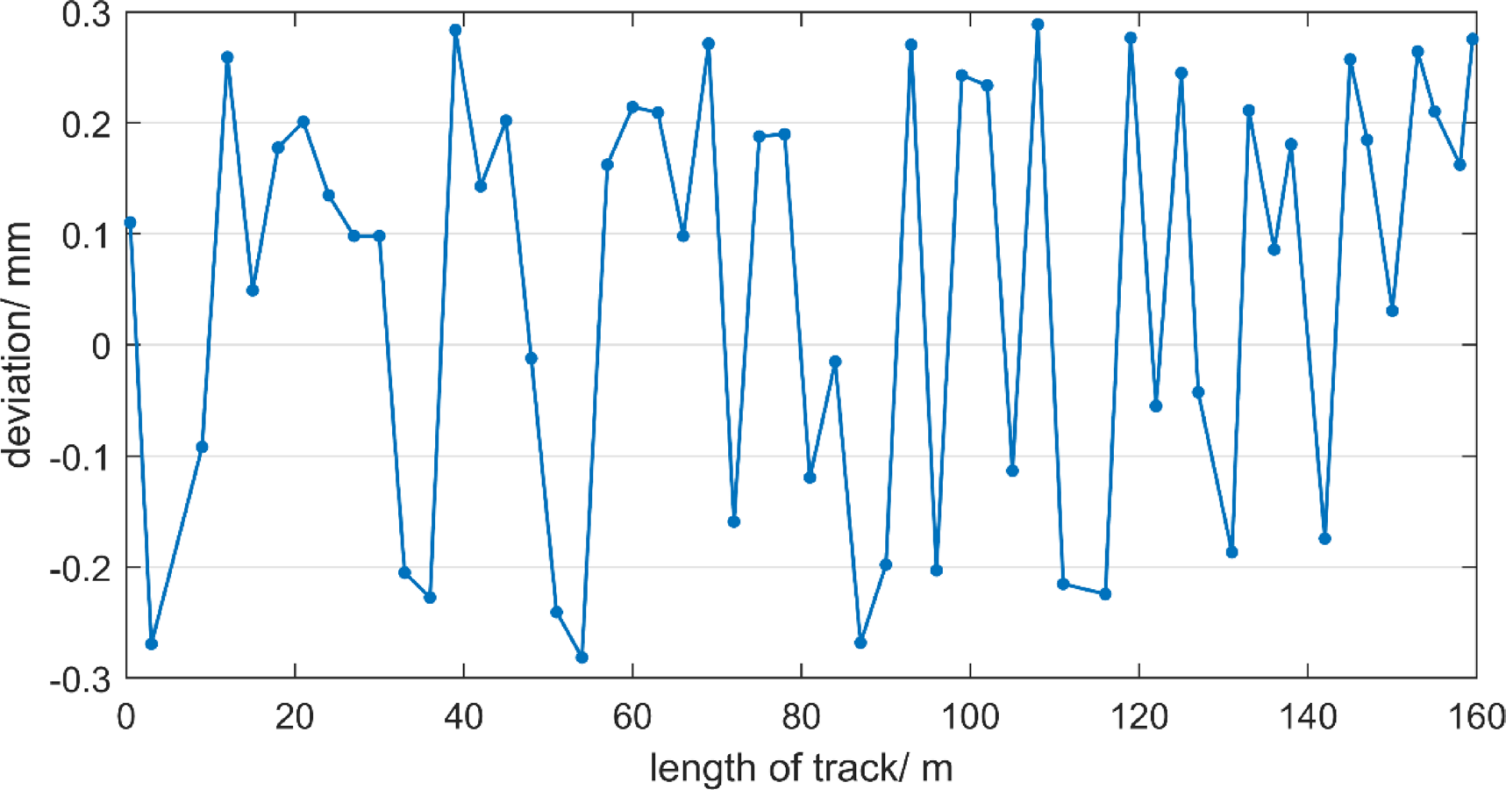
The deviation distribution of each measurement point on the track.

**Fig. 18:**
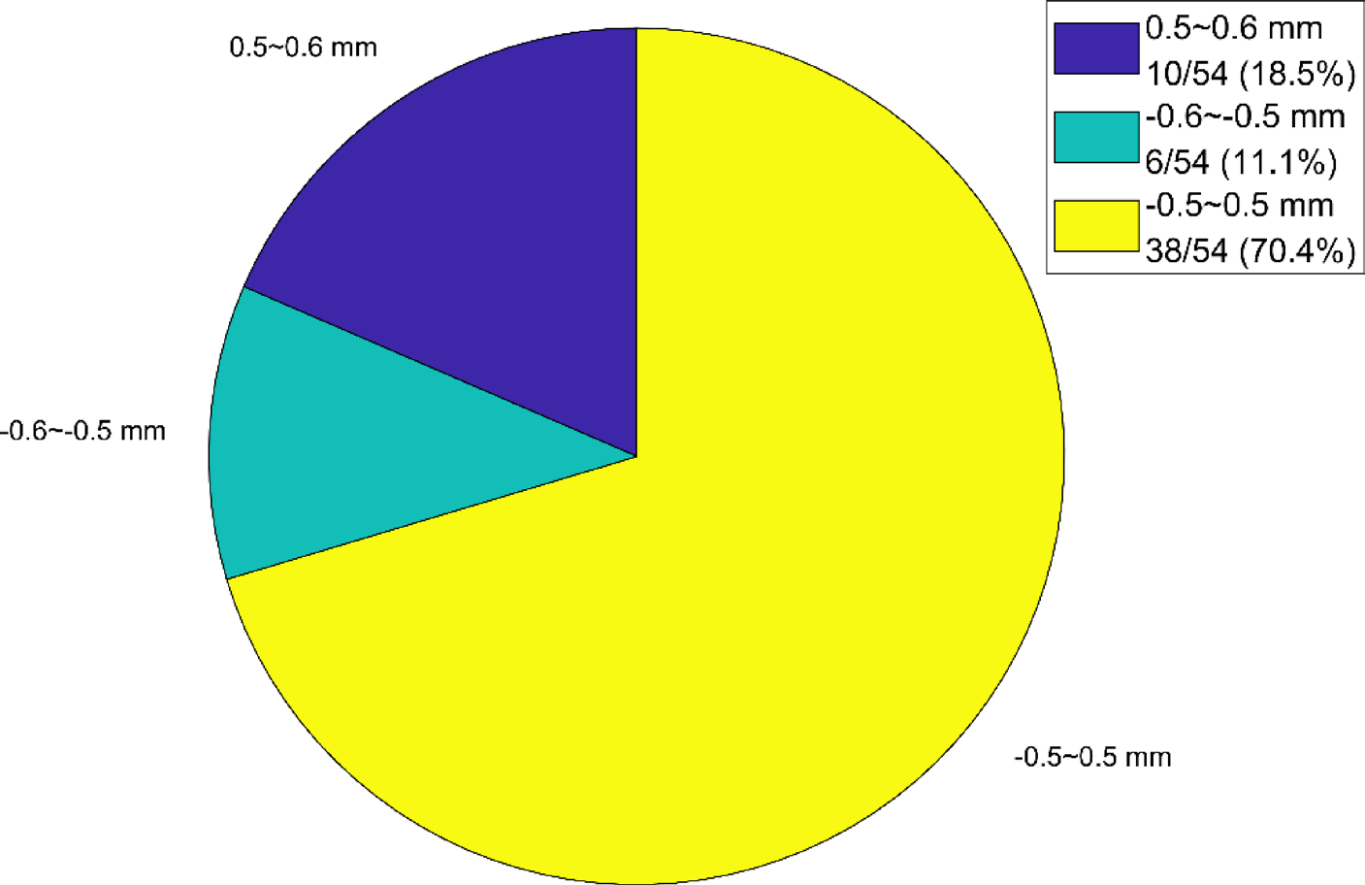
Percentage of the deviation distribution of measurement points on the track considering the mean square error of deviation.

Take the average of the six deviations at each measurement point as the final deviation of each point to the track. And those results are shown in Fig. 17. As can be seen from Fig. 17, the maximum value of the measurement point deviation is 0.29 mm, the minimum value is −0.28 mm, the average value is 0.06 mm, the median is 0.12 mm. The percentage of the deviation distribution of measurement points on the track considering the mean square error of deviation is shown in Fig. 18.

Figure 18 shows that the number of measurement points with a deviation in the range of −0.5 to 0.5 mm is the largest, with 38 points, accounting for 70.4%. Then there are 10 measurement points with a deviation greater than 0.5 mm, accounting for 18.5% and the number of measurement points with a deviation less than − 0.5 mm is the smallest, with 6 points, accounting for 11.1%. Overall, most of the measurement point deviations are concentrated in the range of −0.5 to 0.5 mm, while the proportion of measurement points with a relatively large absolute deviation (less than − 0.5 mm or greater than 0.5 mm) is relatively small.

## Conclusion

The free station method of the total station for straightness measurement is studied, and a nonlinear model for calculating the deviation of measurement points from the baseline using the area of triangles is established. Through systematic Monte Carlo simulation, the influence patterns of angular error, distance error, and instrument station position on deviation measurement accuracy are analyzed. The research finds that the uncertainty of point deviations is symmetrically distributed with the total station as the center, reaching its maximum at the station position, where accuracy is primarily affected by ranging error. For points farther from the total station, accuracy is mainly governed by angular measurement precision. Therefore, selecting a total station with high angular measurement accuracy, such as 0.5 arcseconds, is key to ensuring overall precision, while a distance measurement accuracy within 2 mm is sufficient for long-distance measurement requirements. Observations should be avoided for points located very close (within approximately 20 m) to the total station, where distance measurement error becomes the dominant uncertainty source. By optimizing the total station setup position, specifically by shortening the perpendicular distance from the total station to the baseline and adopting a multi-station segmented observation strategy, the high uncertainty in the near-station area can be effectively controlled, achieving uniform optimization of deviation accuracy along the entire line.

The free station method is applied to measure the straightness of a 160-meter-long track. Using two optimally positioned total stations at 50 m and 100 m respectively, deviations are measured at 54 points. Repeated measurements showed that 70.4% of points (38/54) fall within the ± 0.5 mm tolerance. With a mean square error of approximately ± 0.30 mm and a maximum deviation of 0.29 mm, the measured straightness of the track meets the specified accuracy requirement of ± 0.6 mm.

## Data Availability

The datasets used and/or analysed during the current study available from the corresponding author on reasonable request.
